# Bridging the gap: Translating fetal, infant, and toddler neuroimaging insights into clinical practice

**DOI:** 10.1016/j.dcn.2026.101696

**Published:** 2026-02-17

**Authors:** Paige M. Nelson, Dashiell D. Sacks, Marta Korom, Nushka Remec, Guy A. Perkins, Kevin M. Cook, Sally M. Stoyell, Megan E. Evans, Julia Moser, Chiara Capparini, Sanjana Inala, Istvan N. Huszar, Michal R. Zieff, Angelina Vernetti, Elizabeth S. Norton, Carol L. Wilkinson, Tomoki Arichi

**Affiliations:** a Stead Family Department of Pediatrics, Carver College of Medicine, University of Iowa, Iowa City, IA, USA; b Department of Psychiatry, Carver College of Medicine, University of Iowa, Iowa City, IA, USA; c Department of Psychiatry and Behavioral Sciences, Boston Children’s Hospital, Boston, MA, USA; d Department of Psychiatry, Harvard Medical School, Boston, MA, USA; e National Institute of Mental Health, Bethesda, MD, USA; f Neuroscience Graduate Program, University of Southern California, Los Angeles, CA, USA; g University of Padova, Department of Developmental Psychology and Socialization, Via Venezia, 8, Padova 35131, Italy; h Developing Brain Institute, Children's National Hospital, Washington 20010, DC, USA; i Institute of Child Development, University of Minnesota, MN, USA; j Masonic Institute for the Developing Brain, University of Minnesota, MN, USA; k Fralin Biomedical Research Institute at VTC, Virginia Tech, Roanoke, VA, USA; l Translational Biology, Medicine, & Health Graduate Program, Virginia Tech, Roanoke, VA, USA; m Laboratoire de Neuroanatomie de Neuroimagerie translationnelles (LN2T), ULB Neuroscience Institute (UNI), Université libre de Bruxelles (ULB), Brussels 1070, Belgium; n ULBabyLab, Center for Research in Cognition and Neurosciences (CRCN), ULB Neuroscience Institute (UNI), Université libre de Bruxelles (ULB), Brussels 1050, Belgium; o Department of Psychiatry, Vagelos College of Physicians and Surgeons, Columbia University, New York, NY 10032, USA; p Athinoula A. Martinos Center for Biomedical Imaging, Charlestown, MA 02129, USA; q Department of Radiology, Massachusetts General Hospital, Boston, MA 02114, USA; r Department of Radiology, Harvard Medical School, Boston, MA 02115, USA; s Department of Paediatrics and Child Health, University of Cape Town, Cape Town, South Africa; t Child Study Center, Yale School of Medicine, New Haven, CT, USA; u Roxelyn and Richard Pepper Department of Communication Sciences and Disorders, Northwestern University, Evanston, IL, USA; v Institute for Innovations in Developmental Sciences, Northwestern University, Evanston, IL, USA; w Division of Developmental Medicine, Boston Children's Hospital, Boston, MA, USA; x Harvard Medical School, Boston, MA, USA; y Early Life Imaging Department, School of Biomedical Engineering and Imaging Sciences, King’s College London, UK

**Keywords:** Brain development, Neuroimaging, MRI, Pediatric medicine, Clinical translation

## Abstract

Over the past decade, fetal, infant, and toddler (FIT) neuroimaging has become a rapidly expanding field, driven by advances in technology and computational methods. By providing non-invasive ways to explore the developing brain in both typical and pathological development, FIT neuroimaging holds promise for advancing pediatric medicine. Magnetic resonance imaging (MRI), ultrasonography, and electroencephalography (EEG) are regularly used in clinical practice to identify or rule out structural brain abnormalities; monitor the timing and evolution of brain injuries; assess brain growth and maturation; diagnose and monitor seizures, including infantile spasms; and facilitate pre-surgical planning. Other methods, including functional MRI, magnetoencephalography (MEG), and near-infrared spectroscopy (NIRS), provide information about brain function but have not yet been considered standard-of-care. As the field has rapidly advanced, numerous barriers (e.g., concerns regarding cost-effectiveness, safety, availability and portability, bedside applicability, suitability for serial imaging, and uncertain predictive utility) have hindered the routine use of neuroimaging techniques. This review focuses on how FIT neuroimaging research can be applied beyond academic settings to improve outcomes in clinical environments, such as high-risk follow-up programs and neonatal and pediatric intensive care units. Key topics include: (1) how FIT neuroimaging can advance pediatric medicine; (2) challenges and gaps in translating FIT neuroimaging to clinical practice; (3) proposed strategies for bridging these gaps; and (4) a framework for clinical translation and future directions to enhance pediatric healthcare and developmental outcomes.

## 1 Introduction

Marked advances in technology and computational analysis methodologies have enabled fetal, infant, and toddler (FIT) neuroimaging to emerge as a rapidly expanding and independent research domain. FIT neuroimaging provides an unprecedented wealth of new knowledge about how brain structure and function develop in the critically important first years following birth (Vaughn et al., 2023). This period is among the most dynamic and influential phases of human neurodevelopment (Knickmeyer et al., 2008), with total cerebral volume increasing by 230 % between 25 and 36 weeks of gestation and the cerebellum expanding by 384 % (Clouchoux et al., 2012). This is followed by postnatal expansion, as the brain reaches roughly 64 % of its adult volume by 90 days after birth (Holland et al., 2014), with fundamental macroscopic neuroarchitecture and functional organization established by two years of age (Dubois et al., 2014, Sampaio and Truwit, 2001). This rapid development likely renders the developing brain highly vulnerable to intrinsic or extrinsic insults during the prenatal and postnatal periods, including genetic factors, adverse events, or acquired pathology (Fei et al., 2014, Tataranno et al., 2021, Volpe, 2019). Conversely, this early window of rapid development may also present potential opportunities for increased sensitivity to clinical interventions and treatments due to the enhanced neuroplasticity in this period (Knickmeyer et al., 2008, Matthews et al., 2018).

**Fig. 1:**
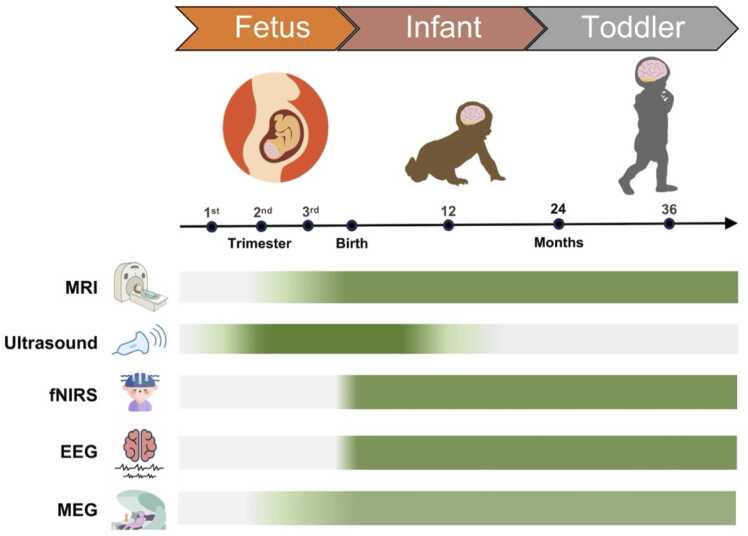
Applicability of neuroimaging modalities across early developmental stages. The timeline highlights three developmental periods (fetus, infant, and toddler) alongside key age milestones. Horizontal bars represent the applicability of a select list of neuroimaging modalities, including magnetic resonance imaging (MRI), ultrasonography, functional near-infrared spectroscopy (fNIRS), electroencephalography (EEG), and magnetoencephalography (MEG), with darker shading indicating greater feasibility for use at a given age. Ultrasound is the most feasible during the fetal and early neonatal stages, whereas modalities such as MRI, MEG, fNIRS, and EEG become increasingly suitable from infancy through toddlerhood.

By providing a non-invasive means by which to visualize and study dynamic changes in early brain structure and function during both healthy development and pathological states, FIT neuroimaging has clear potential to advance pediatric medicine. Such approaches may help inform and refine individualized patient care plans through enhanced diagnosis and prognosis, and as the field continues to evolve, pave the way toward future precision-focused interventions. Neuroimaging modalities, such as magnetic resonance imaging (MRI), ultrasonography, and electroencephalography (EEG), have now firmly established themselves as standard of care in clinical practice, where they are regularly used to identify and/or rule out structural brain abnormalities; monitor the timing and evolution of brain injuries; assess brain growth and maturation; diagnose and monitor seizures andinfantile spasms, and even facilitate pre-surgical planning (Counsell et al., 2019). In addition, several other methods now widely used in neuroscience and psychological research, including functional MRI (fMRI), magnetoencephalography (MEG), and near-infrared spectroscopy (NIRS), can also provide information about brain function and how it relates to behavior and later cognition (Fig. 1). However, as the field has rapidly advanced, many of these innovations have not been translated into regular clinical use because of a combination of barriers and challenges. These include concerns regarding cost-effectiveness; safety (e.g., exposure to ionizing radiation and potential heating related to specific absorption rate [SAR] during MRI acquisitions); availability and portability; bedside applicability; suitability for serial imaging; and uncertain predictive utility, given their low sensitivity and specificity for long-term developmental outcomes (El-Dib et al., 2023a, El-Dib et al., 2023b, Inder et al., 2003, Inder et al., 2021). Furthermore, other prominent factors include geographical disparities in infrastructure, training, and access to these neuroimaging modalities, particularly in low- and middle-income countries (Geethanath and Vaughan Jr., 2019; Luntsi et al., 2021; Ogbole et al., 2018), as well as variations in standard-of care-protocols (Austin et al., 2024).

In order to understand the current state-of-the-art techniques and formulate research questions that can meaningfully impact pediatric clinical care while cultivating collaborative relationships with clinicians, a group of attendees at the FIT’NG 2024 annual conference held a reflective discussion, which is presented in this review. We focused on how FIT neuroimaging research and insights can be applied beyond academic settings to enhance outcomes in real-world clinical environments, such as within high-risk follow-up programs and neonatal and pediatric intensive care units. The key topics we consider are: how FIT neuroimaging research can advance pediatric medicine; the challenges and gaps in translating FIT neuroimaging research to clinical practice; proposed strategies for bridging these gaps and addressing the challenges of applying and implementing FIT neuroimaging research and insights into clinical application; and a framework for translation and future directions aimed at enhancing pediatric healthcare and developmental outcomes in real-world settings. In addition, we introduced a set of self-reflective questions intended to guide researchers and clinicians when designing or interpreting studies, with the goal of better scaffolding the translational relevance of FIT neuroimaging findings to everyday clinical practice (Table 1).

**Table 1:** Key self-reflective questions to guide FIT neuroimaging researchers when designing studies.

**Developmental Modeling and Incremental Prediction** 1. When analyzing longitudinal data, does my analysis plan model nonlinear and/or heterogeneous developmental trajectories (e.g., timing shifts, slope differences, or stable variance)? Have I examined whether these patterns differ by relevant moderators (e.g., age, biological sex, nature of brain insult, socioeconomic status/environmental factors, genetics if/when available)? 2. When modeled alongside key demographic, biological, medical, and environmental determinants of outcome, do my neuroimaging markers demonstrate an independent association with the outcome? If so, what proportion of unique variance does it explain beyond these covariates? 3. Do my neuroimaging measures provide incremental predictive utility for individual-level prognosis beyond standard clinical indicators, as assessed by metrics such as sensitivity/specificity, receiver operating characteristic (ROC) curves, or likelihood ratios?
**Methodological Rigor and Reproducibility** 4. Are my neuroimaging modalities, sequences, and scan timing choices justified in light of neurodevelopmental timing and clinical feasibility? Have I clearly explained why I selected specific techniques (e.g., DWI, 1H-MRS), sequences (e.g., T1/T2 vs. ADC), and scan windows (e.g., early injury detection vs. later severity classification), and acknowledged their clinical trade-offs? 5. Have I aligned my data acquisition and processing protocols (e.g., hardware model, sequence parameters, preprocessing pipelines, QC procedures, software versions) with any established or published standards/frameworks (e.g., BCP, INTERGROWTH-21st)? If not, do I transparently report decisions regarding deviations that may impact reproducibility across sites, samples, and developmental stages (e.g., QC, motion thresholds, scoring protocols)? 6. Are my processing pipelines and analysis workflows publicly accessible/open-source (e.g., via pre-registration, OSF, GitHub repositories)? Are they designed (or adapted) to handle age-specific challenges in FIT populations (e.g., using HAPPE, MADE, fMRIPrep Lifespan)? 7. In the case of multisite studies, have I discussed how methodological differences, such as the modality-specific hardware (e.g., EEG systems, MRI field strengths, head coil choice, US transducers), and data analysis tools/pipelines introduce variability and/or limit cross-site data harmonization?
**Representativeness and Scalability** 8. Does my sample adequately reflect the demographic characteristics (e.g., race, ethnicity, socioeconomic status, geography) and clinical diversity (e.g., preterm birth, congenital heart disease) of the population of interest? 9. Is my study’s proposed methodology operationally feasible and scalable across various clinical settings (e.g., NICUs, rural clinics) with limited staff and/or infrastructure? Is it cost-effective and deployable in low-resource or remote settings (e.g., facilities without stable electricity or internet access)?
**Clinical Integration and Implementation** 10. Have I meaningfully engaged clinical stakeholders in designing, interpreting, and disseminating my work? If yes, have I ensured that clinician participation is logistically and financially supported? If these responsibilities extend beyond clinicians’ established roles, does the study introduce unintended workflow disruptions or uncompensated workload demands (e.g., recruitment, data collection, interpretation)? 11. Have I considered and reported on the practical steps needed to implement my study methodology and imaging tools used in my study in clinical care (e.g., cost, staff training, maintenance of equipment, and compatibility with clinical systems) and evaluated whether the benefits justify these investments?

## 2 How can FIT neuroimaging research advance pediatric medicine?

### 2.1 Examples of FIT neuroimaging practices that are currently standard of care

There are specific examples where routine neuroimaging has become standard of care in pediatric medical settings, notably in cases where it has an invaluable role in diagnosis, prognosis, and guiding individualized care which may eventually support precision-focused interventions. This includes the widespread use of EEG for diagnosing and monitoring seizures, including infantile spasms, and MRI and ultrasonography for identifying structural brain injuries, such as areas of infarction, hemorrhage, and brain tumors in pediatric populations presenting with neurological symptoms. In addition to these well-established applications, there are developmental contexts in which FIT neuroimaging has also become integrated into standardized screening and monitoring workflows, particularly in prenatal and neonatal intensive care settings.

For instance, the American College of Obstetricians and Gynecologists and the UK National Health Service Fetal Anomaly Screening Program both recommend fetal transabdominal ultrasound as the standard-of-care screening around 18–22 weeks of gestation to assess fetal head biometry (e.g., biparietal diameter), anatomical integrity (e.g., cerebral hemispheres, ventricles, and cerebellum), and congenital anomalies of the central nervous system (Bulletins—Obstetrics and Medicine, 2016, Milani et al., 2019, Napolitano et al., 2016, Napolitano et al., 2020, NHS England, 2024). When fetal ultrasound is inconclusive due to poor visualizations and/or congenital brain anomalies, fetal MRI may be warranted to provide more detailed assessment of fetal brain anatomy and reduce diagnostic uncertainty (Alluhaybi et al., 2022, Chaudhari and Ho, 2022, Manganaro et al., 2017, Nagaraj and Kline-Fath, 2022, Powers et al., 2022).

Similarly, most professional pediatric organizations, including the American Academy of Pediatrics and Canadian Pediatric Society, have guidelines for standard intervals and imaging protocols for routine clinical ultrasound screening following preterm birth (< 37 weeks of gestation) in the neonatal intensive care unit (NICU) (Canadian Paediatric Society, 2001, Hand et al., 2020). For those born ≤ 30 weeks of gestation, these typically include ultrasound scans in the first 2 weeks following birth, with repeat scans every few weeks during NICU admission, and a concluding scan around term-equivalent age (36 weeks postmenstrual age) or prior to hospital discharge (Inder et al., 2021). The initial ultrasound examination assesses early germinal matrix–intraventricular hemorrhages, whilst subsequent exams monitor the progression of insults and identify new lesions, such as ventriculomegaly resulting from white matter loss and periventricular leukomalacia (Meijler and Steggerda, 2019). Notably, ultrasound findings provide clinicians valuable insights into determining optimal treatment and intervention strategies, particularly in cases of excessive buildup of cerebrospinal fluid in the ventricular system (Dutch Working Group of Neonatal Neurology, 2002, Leijser et al., 2018, Sewell et al., 2025).

Another context in which neuroimaging has been integrated into routine clinical care is in the evaluation and management of neonatal encephalopathy, including hypoxic-ischemic encephalopathy (HIE) resulting from perinatal asphyxia (El-Dib et al., 2023a, El-Dib et al., 2023b). Both EEG and MRI play central roles in the diagnosis, prognostication, and ongoing management of infants with HIE. In the NICU, continuous EEG monitoring has become increasingly standard practice, with amplitude-integrated EEG (aEEG) – a time-compressed display of filtered cerebral electrical activity from a subset of electrodes – commonly displayed at the bedside, particularly with infants undergoing therapeutic hypothermia (El-Dib et al., 2009, Shah and Wusthoff, 2015). Classification systems of this simpler aEEG facilitate the detection of clinical and subclinical seizures by trained nurses and providers who score the aEEG based on patterns of activity (e.g., continuous, discontinuous, burst suppression, low-voltage, flat-line) (Burdjalov et al., 2003, Hellstrom-Westas et al., 2006). This may then be combined with specialist review of continuous EEG data and MRI for prognostication and counseling. MRI is usually performed between 5 and 14 days after birth (Austin et al., 2024) rewarming from therapeutic hypothermia, in order to define injury patterns and inform prognosis. Standard protocols for HIE typically include diffusion-weighted imaging (DWI), T1- and T2-weighted scans, and proton magnetic resonance spectroscopy (1H-MRS) to measure the deep grey matter lactate to N-acetylaspartate ratio which have been found to significantly predict outcomes (Parmentier et al., 2022, Weeke et al., 2018). Together, these practices demonstrate that FIT neuroimaging can be successfully integrated in clinical settings, while also setting the stage for strategies to enhance its impact based on evidence from both research and clinical experience.

### 2.2 Potential of FIT neuroimaging for wider clinical applications

Recent technological innovations, ranging from advances in FIT neuroimaging hardware to enhanced computational power and more sophisticated analytic tools, are improving existing clinical neuroimaging methods, protocols, and techniques. These innovations, for example, may enhance signal-to-noise ratio (e.g., custom-designed MRI receiving coils) and enable higher spatial and temporal resolution, supporting more accurate diagnosis and monitoring of neurological injuries and disorders (Dubois et al., 2021, Stout et al., 2021). Importantly, they are also supporting the integration of advanced neuroimaging methodologies, that have historically been confined to research settings (e.g., fMRI, DTI, MEG), into clinical standards of care.

Advances in FIT neuroimaging can also play an important role in pediatric medicine by enabling the development of novel biomarkers for early diagnosis and improved prediction of later outcomes, thereby supporting earlier risk detection, targeted intervention, and monitoring of disease progression. For instance, studies have identified potential biomarkers of post-hemorrhagic hydrocephalus, such as DWI measures of abnormal white matter microstructure in the perimeters of the lateral ventricle frontal-occipital horns (Isaacs et al., 2022). Their predictive utility may be substantially enhanced when integrated with other clinical, biological, and environmental factors, rather than considered in isolation. These factors include, but are not limited to, neurological abnormalities, neonatal morbidities/interventions (e.g., bronchopulmonary dysplasia, postnatal corticosteroids), socioeconomic status, caregiving environment, and genetic/epigenetic influences. Together, this integrative approach may help identify infants who could benefit from early enrollment in high-risk infant follow-up programs, while ultimately informing the development and implementation of novel interventions (Anderson et al., 2015, Guillot et al., 2020, Machie et al., 2024; Nelson et al., 2021; Nikolaeva et al., 2024; Parikh, 2016). This is important, as current evidence suggests that FIT neuroimaging abnormalities alone are not definitive predictors of severe neurodevelopmental impairments, nor are neurotypical brain imaging scans guarantees of appropriate development (Edwards et al., 2018, for the SUPPORT Study Group of the Eunice Kennedy Shriver National Institute of Child Health and Human Development Neonatal Research Network, 2015, Woodward et al., 2006).

FIT neuroimaging can also play a role in guiding clinical decision-making and counseling of families, particularly when assessing the continuation or adjustment of neonatal or pediatric intensive care for high-risk neonates and infants with moderate-to-severe brain injuries (Hinojosa-Rodríguez et al., 2017, for the SUPPORT Study Group of the Eunice Kennedy Shriver National Institute of Child Health and Human Development Neonatal Research Network, 2015, Inder et al., 2021, Meijler and Steggerda, 2019). For instance, studies have consistently found that 80 % of germinal-matrix intraventricular hemorrhage occurs in preterm infants within the first 72 h after birth (De Vries et al., 2015). Additionally, around 30–50 % of infants with a large intraventricular hemorrhage later develop post-hemorrhagic ventricular dilatation, and approximately 20–40 % of these infants are subsequently diagnosed with post-hemorrhagic hydrocephalus requiring insertion of a permanent ventriculo-peritoneal shunt (Adams-Chapman et al., 2008, Klebermass-Schrehof et al., 2013). This highlights the need for future prospective studies to investigate the optimal timing of neurosurgical intervention.

In addition, FIT neuroimaging can be used to quantitatively evaluate the effectiveness of interventions and thus provide evidence for their possible implementation into standard clinical care (Papatzikis, 2024). For example, auditory experiences in the NICU environment are vastly different from those in the womb (DeMaster et al., 2019, Flake, 2022, Matthews et al., 2018, McGowan and Vohr, 2019). This is particularly relevant for preterm infants as they lose the acoustic modulation of the womb, thus experiencing near continuous high-frequency and high-volume noise through air conduction, ranging from 500 to 16,000 Hz (Lahav, 2015). fMRI studies suggest that enriching the NICU environment with repeated music listening can modify functional connectivity between auditory cortices, thalamus, and dorsal striatum in preterm infants, such that connectivity between these sensory regions is less disrupted and more closely mirrors patterns observed in term born infants (e.g., Lordier et al., 2019). Importantly, there is still a paucity of longitudinal studies characterizing more distal impacts on long-term development, so it is not clear how durable these changes are beyond infancy. Nonetheless, FIT neuroimaging has potential to significantly influence neonatal and pediatric medicine, enabling clinicians to make more informed, proactive, and refined decisions regarding the diagnosis, treatment, and long-term management of high-risk individuals. As longitudinal cohorts are followed into childhood and adolescence, even more robust findings will be developed that can inform care to maximize development well into the lifespan.

## 3 Challenges and gaps in translating fetal, infant, and toddler neuroimaging research to clinical practice

While FIT neuroimaging is already used in select clinical contexts and shows promise for advancing pediatric medicine, substantial barriers still exist for translating FIT neuroimaging methods more broadly. A snapshot of these translational challenges is discussed below.

### 3.1 Variability in developmental trajectories and limitations of prediction and interpretation limitations

Perhaps one of the greatest challenges in translating clinical findings into actionable, idiographic goals is the marked variability in developmental trajectories in early childhood (Batalle et al., 2018), even among individuals with similar neuroimaging profiles and/or those considered neurotypical. For instance, cortical volume or white matter integrity may appear preserved on neonatal MRI scans even in infants who later show diverging outcomes following mild or moderate-to-severe brain injuries. This variability not only arises from the nature of brain insults (e.g., differences in age at injury, severity, and anatomical extent), but can also stem from a wide range of environmental (e.g., family context, socioeconomic status, exposures to different language, social, or sensory experiences) and genetic influences that shape neuroplasticity and modulate the timing of sensitive developmental periods (Catroppa et al., 2008, Luciana, 2003, Olson et al., 2025).

As such, the magnitude of individual differences in complex developmental pathways often masks the subtle effects that researchers aim to detect, which in turn can limit statistical power and interpretability. For example, these individual differences can manifest as shifts in the timing of developmental phases (Fuhrmann et al., 2022) or highly variable and nonlinear growth trajectories (Sanders et al., 2023). Even among FIT cohorts considered neurotypical, parsing this developmental complexity remains an ongoing endeavor, as many researchers have experienced firsthand when longitudinal patterns fail to meet expectations (Mills et al., 2021). Consequently, establishing robust normative baselines against which clinically meaningful deviations can be accurately interpreted remains a considerable challenge for the field.

In everyday research and clinical settings, especially where a variety of FIT neuroimaging hardware and sophisticated analytical tools are used, these confounding factors often interact in non-deterministic ways, further complicating the challenge of predicting both short- and long-term developmental outcomes (Batalle et al., 2018). Variability in results across studies and heterogeneity within individuals, where the presence of significant group-level trends nonetheless lacks predictive specificity at the individual level, contribute to small effect sizes and wide confidence intervals. This also potentially impacts sensitivity and undermines the clinical utility of FIT neuroimaging research methods, as clinicians need accurate, empirically-derived information, often expressed as binary indicators (e.g., below vs above a clinical risk threshold), to inform individual-level practice (Anderson et al., 2017, Kelly et al., 2019).

The challenge of variability in predictive utility is further illustrated in a systematic review of 47 studies examining the association between advanced MRI biomarkers and neurodevelopmental impairment in very preterm infants (≤ 32 weeks of gestation) or those deemed very low birth weight (< 1500 g) (Parikh, 2016). Although most studies found at least one novel brain marker (derived from DWI, MRS, fMRI, quantitative brain morphometry, and lesion metrics) to be a strong predictor of neurodevelopmental impairments or challenges, including cerebral palsy, cognitive or intellectual impairments, and behavioral or psychological diagnoses such as autism and ADHD, there was substantial heterogeneity across studies. Numerous contributing methodological limitations were highlighted, including variable sample sizes (ranging from 10 to 297 infants), with most having fewer than 100 infants. Additionally, around one-third of the studies failed to evaluate the incremental predictive value of significant biomarkers over known clinical indicators (such as visualization of insults on structural MRI), with the majority not reporting diagnostic metrics, including sensitivity, specificity, or likelihood ratios (Parikh, 2016).

Prediction of developmental outcomes at two years is also highly variable in studies of term-born HIE neonates treated with therapeutic hypothermia (Andorka et al., 2024, Estiphan et al., 2024, Wu et al., 2023). This inconsistency persists despite the development and standardization of numerous MRI-based scoring systems for assessing perinatal asphyxia (Barkovich et al., 1998, Rutherford et al., 1996, Shankaran et al., 2012, Weeke et al., 2018). Part of this challenge relates to sensitivity to different aspects of the evolving pathology, with early MRI better capturing the acute brain injury, while later MRI is more effective at assessing the extent and severity of the injury. Different MRI acquisitions also have sensitivity to specific aspects of the evolving brain injury. For example, apparent diffusion coefficient (ADC) measures derived from DWI are more sensitive to brain abnormalities in the first days following injury, whereas T1/T2-weighted changes become more evident the end of the first week, with the extent of the injury clearer by the second week (Weeke et al., 2018).

### 3.2 Barriers to accessible and universal deployment of FIT neuroimaging

The integration of FIT neuroimaging insights into routine clinical practice is meaningfully affected by the systemic, financial, and infrastructural disparities that persist within and across global healthcare systems and societies (Anindya et al., 2021, Slopen et al., 2024). For instance, healthcare systems are affected by primary structural limitations, such as fragmentation of public and private sectors, inconsistent or insufficient insurance coverage, and underfunded government initiatives which can exacerbate divides relating to the location and socio-economic status of countries and families. The cost of acquiring and maintaining high-field strength MRI systems is substantial, and thus they are far less common in low- and middle-income countries. The USA has one MRI scanner per ∼25,000 inhabitants, whereas countries in Southeast Asia and Sub-Saharan Africa have more than 50 times as many residents per MRI scanner (Deoni et al., 2021). In these countries, funding for training healthcare and imaging professionals, and healthcare in general, further hinders the ability to implement FIT neuroimaging tools at scale and thus their potential to be integrated into routine clinical practice (Duncan and Rae, 2024, Geethanath and Vaughan, 2019).

Even for ultrasound, a more widely used FIT neuroimaging modality which is lower cost and more portable compared to MRI (Tang et al., 2022), disparities remain stark. Most women in developing regions, like sub-Saharan Africa, lack or have limited access to even a single ultrasound throughout their pregnancy (Aliyu et al., 2016, Luntsi et al., 2021, Wanyonyi et al., 2017). This limited access increases pregnancy risks and contributes to higher rates of delivery complications, perinatal mortality, and neonatal morbidities (Sendra-Balcells et al., 2023). This is partly due to financial constraints, the need to travel long distances to access essential medical care, and the scarcity of trained sonographers and specialized sonography-trained clinicians, even in urban areas (Nathan et al., 2017).

Those in under-served, poorer, and more rural areas globally are not only less able to obtain clinical neuroimaging when required, which affects their health care, but also means that substantial populations are under- or un-represented in neuroimaging research (Aderinto et al., 2023). Even in high-income countries like the UK and USA with greater access to advanced neuroimaging, inequalities persist, particularly among vulnerable and underserved populations (Deoni et al., 2021). The fiscal and cultural inequities within and across countries mean that studies predominantly involve populations of European descent located in populated areas. Because clinical research studies do not include all relevant populations across regions and groups, this limits clinical utility and further perpetuates inequities in pediatric healthcare (Margolis and Nelson et al., 2025). The barriers to and need for including globally representative samples in FIT neuroimaging research and how they vary by modality are described in a FIT'NG conference-facilitated paper by Margolis & Nelson et al. (2025). These same factors affect how such neuroimaging information can be incorporated in clinical practice. Without substantial policy changes and resource reallocation, equitable access to neuroimaging technologies across different healthcare settings remains a distant and unlikely goal.

Even in high-income countries, research involving clinical patients, particularly critically ill cohorts in the NICU or pediatric intensive care unit (PICU), is often limited to measures that are easily accessible in clinical environments, like conventional field strength (1.5 or 3 T) MRI (Barkovich et al., 2019). This is typically coupled with clinicians' qualitative assessment of structural scans and sometimes complemented by categorical scoring systems, as seen in manual HIE MRI scoring (Barkovich et al., 1998, Weeke et al., 2018). More sensitive neuroimaging techniques, such as volumetric MRI, MRS, DWI, and fMRI, that can focus on measuring specific mechanisms, such as metabolic derangements, myelination, or functional connectivity (Anderson et al., 2015, Parikh, 2016, Tao and Neil, 2014), are typically less accessible in clinical settings. This limitation is particularly evident in clinical groups with overt structural brain injury, such as perinatal stroke, where there are also likely marked alterations in functional connectivity and/or regional function (Domi et al., 2017).

### 3.3 Methodological variability in acquisition and processing

A further major challenge comes through the variability in data acquisition protocols and pre- and post-processing techniques across different neuroimaging modalities, research sites, and studies. Substantial heterogeneity can contribute to inconsistencies in data quality, interpretation, and generalizability of results. For instance, there is variability in EEG hardware (e.g., EEG system, number and layout of electrodes) and signal processing methods (e.g., preprocessing, processing parameters) (Bigdely-Shamlo et al., 2015, Croft and Barry, 2000, Gabard-Durnam et al., 2018, Luck, 2014, Troller-Renfree et al., 2025). Similarly, postnatal ultrasound data are variable because of differences in machines, transducer types and frequencies (Meijler and Steggerda, 2019, Ecury-Goossen et al., 2015, van Wezel-Meijler and de Vries, 2014), acquisition parameters, and operator technique, with many centers relying on manual measurements by professionals with varied training. These inconsistencies are compounded by variable definitions regarding boundaries of neonatal brain regions (Cuzzilla et al., 2018, Fox et al., 2014). Likewise, variability in MRI arises from differences in field strength (e.g., 1.5 T versus 3 T), imaging sequences, and preprocessing pipelines, which are often selected based on individual experience rather than established standards (Glasser et al., 2013, Glasser et al., 2016, Van Essen et al., 2013). Across modalities, these issues can be exacerbated among high-risk FIT populations, where anatomical structures are immature and acquisition can be more challenging, leading to increased variability in methods and outcomes.

Despite early efforts toward harmonization, substantial methodological variability persists because of the lack of consensus on best practices and the competing demands of flexibility versus standardization. For example, approaches such as transitioning from manual to automated or semi-automated processing pipelines can support standardization and reproducibility, which is further discussed in Section 4.1. However, the efficacy of this strategy depends on broad consensus, coordinated field-wide harmonization efforts, and consideration of the frequently competing priorities of academia and industry. In fact, although automated preprocessing pipelines can serve as a tool to improve consistency, when separate automated pipelines are developed in parallel without harmonization, their proliferation can paradoxically hinder standardization efforts and reinforce methodological inconsistencies across studies. Empirical data on pipeline or toolbox re-use within the FIT community remains limited, making it difficult to determine which processing steps should be standardized and which should remain flexible. Furthermore, many research pipelines are designed for users with substantial domain expertise in processing methods, software, and coding. In contrast, some of the strongest incentives for standardization come from profit-driven commercial competition, with companies developing clinically oriented tools that provide standardized acquisition sequences and streamlined processing software. Particularly in the context of harmonizing tools across research and clinical use, stronger industry collaborations might be necessary to build commercially available, easy to use tools that will be maintained long term and can be deployed across both research and clinical settings.

### 3.4 Structural and systemic discrepancies in clinical vs. research settings

Several structural and systemic factors contribute to the gap between scientists and clinicians, driven by differences in training, priorities, and career trajectories, which can ultimately lead to divergent priorities and reduced opportunities for meaningful collaboration. Notably, FIT neuroimaging researchers are typically trained and incentivized to prioritize scientific inquiry and advance the field’s mechanistic understanding of early neurodevelopment through rigorous study design, hypothesis testing, and analytical innovation (Pollatou et al., 2022). Academic reward structures generally reinforce this through the need to secure competitive funding, publish in high-impact journals, teach, mentor, and service to the wider field, many of which are only indirectly related to clinical translation. In contrast, clinicians are typically trained and rewarded for providing effective, time-sensitive care to pediatric patients, and clinical training places emphasis on the application of established evidence over and above the generation of new knowledge and innovation. Furthermore, the demands of clinical practice, such as high patient volumes, administrative burdens, limited resources and funding, and stringent time constraints, can leave clinicians limited time to deeply engage and collaborate in translational research (Kelly et al., 2019). Importantly, while there are undoubtedly excellent clinicians engaging in research and clinician-researcher collaborations, the aforementioned structural and systemic factors continue to limit more widespread adoption of integrated clinical research.

Another major misalignment arises when the clinical return on investment becomes uncertain, because the substantial time and workflow/billing changes required from adopting research-driven FIT neuroimaging methods are only justified when those methods are sensitive, specific, and practical for clinical use (Atsina et al., 2020, Cocozza et al., 2016). Until research-driven FIT neuroimaging methods are widely perceived as justified and worthwhile through offering meaningful, actionable clinical insights, the paths of FIT neuroimaging research and clinical practice will likely continue to increasingly diverge (Paget et al., 2017). Each branch may remain in its own silo, limiting opportunities for cross-disciplinary engagement and shared learning (Gelijns and Gabriel, 2012, Paquette et al., 2025), ultimately hindering the translation of promising FIT neuroimaging research into everyday clinical practice.

## 4 Proposed strategies for bridging the gap

Researchers are beginning to make headway in overcoming the significant challenges and gaps in translating FIT neuroimaging research to clinical practice, and these, along with a snapshot of further proposed strategies more broadly, are discussed below.

### 4.1 Standardization and creating open neuroimaging protocols

For FIT neuroimaging research and insights to be integrated into clinical practice and utilized as diagnostic instruments, they must be robust and openly available. For instance, attempting to standardize neuroimaging data acquisition protocols and study designs across different geographical locations and studies can help establish the reliability of findings (i.e., replication) and create a robust evidence base of the results necessary for future translation into clinical practice. This is especially critical in FIT populations, where dynamic changes in brain development often require age-specific processing approaches (Goncalves et al., 2025, Hendrickson et al., 2025).

Recent methodological efforts have begun to standardize neuroimaging procedures in FIT research across commonly used modalities, including EEG (Buzzell et al., 2023, Troller-Renfree et al., 2025), MRI (Dufford et al., 2022), fNIRS (Gemignani et al., 2023), and MEG (Clarke et al., 2022). Large-scale, multisite studies, such as the Healthy Brain and Child Development (HBCD; Nelson et al., 2024) study, the Baby Connectome Project (BCP; Howell et al., 2019), and the International Fetal and Newborn Growth Consortium for the 21st Century (INTERGROWTH-21st) Fetal Growth Longitudinal Study (Villar et al., 2013), are designed to address these issues by collecting data with harmonized protocols across multiple national and/or international sites, along with mitigating biases associated with small samples and limited diversity.

Many of these studies have published their protocols as open-source resources (e.g., HBCD EEG protocol: Fox et al., 2024; BCP protocol: Howell et al., 2019). Thus, one strategy may be to harmonize or align protocols in smaller studies with specific research questions, or those involving underrepresented populations, with larger-scale studies. This can facilitate the comparison of results and, in some instances, data pooling, while also increasing cost efficiency (e.g., avoiding the need to recruit new participants for comparison groups).

More generally, the broader adoption of open science practices, including study preregistration and protocol sharing, can further enhance the transparency, reproducibility, and clinical applicability of findings from FIT neuroimaging. In cases in which sharing protocols across multiple labs might be challenging, such as for rare neurological disorders or lab-specific advanced neuroimaging methods, self-replication can be another important tool. For instance, study findings can be replicated in a new sample or with a slightly different neuroimaging method or technique to build on previous work. Such practices establish reliability of research findings and facilitate clinical transfer by building trust in a given finding. While platforms like the Open Science Framework (Foster and Deardorff, 2017) already support preregistration and protocol sharing, an embedded or distinct FIT-specific centralized registry could provide complementary guidance tailored to the unique challenges of FIT neuroimaging research, such as age- and modality-specific protocols and experimental design considerations. Such a resource could promote transparent documentation of protocols and methods, push towards harmonization and consensus, and ultimately support the replicability and generalizability of findings, thereby fostering meaningful collaborations across the FIT neuroimaging community.

In addition to sharing protocols, it is essential for FIT neuroimaging researchers to share practical advice and educate one another on how to implement these protocols effectively, particularly while considering various developmental contexts (e.g., specific age groups, preterm vs. full-term), an effort that should be further incentivized within the field. For instance, Behm and colleagues (2025) provide a thought-provoking example of how to leverage and combine multiple datasets to successfully share practical recommendations on awake infant fMRI recruitment, experimental design, and data acquisition to encourage and optimize future awake infant fMRI research. Emphasizing these practical considerations will increase the feasibility of using these strategies in a clinical context, depending on the intensity of these protocols in terms of time commitment and staffing requirements. This could, for instance, eventually be summarized in a clinical feasibility score assigned to a protocol, which would provide an estimate of how far a method is from being implemented in a clinical setting.

To produce robust and reproducible findings for clinical translation, it is also essential to consider data processing, as even slight variations in pre-processing methods can lead to inconsistent findings across studies (Li et al., 2024). For example, community adaptation of standardized and containerized pipelines like fMRIPrep (Esteban et al., 2019) for structural and functional MRI processing can help mitigate this problem. Standardized analytical frameworks and open-source toolkits enhance reproducibility by ensuring that methodologies can be replicated across different studies. Many standardized processing pipelines are not built explicitly for FIT neuroimaging data; however, this is a rapidly developing field. A few key examples of standardized approaches currently in use for FIT-specific neuroimaging data include fMRIPrep Lifespan (NiBabies; Goncalves et al., 2025) for infant fMRI, Infant Freesurfer (Zöllei et al., 2020) for MRI surface reconstruction, and Harvard Automated Processing Pipeline for Electroencephalography (HAPPE; Gabard-Durnam et al., 2018) and Maryland Analysis of Developmental EEG (MADE; Debnath et al., 2020) for EEG. These standardized pipelines are yet to be specifically developed in NIRS, wherein the field currently utilizes tools not specifically developed for newborn and infant fNIRS data and analysis, such as Homer (Huppert et al., 2009), NIRFAST (Dehghani et al., 2009), and NeuroDot (Speh et al., 2024). Furthermore, the adaptation of a scoring system, including coding infrastructure, testing and benchmarking procedures, and documentation, for software used in FIT neuroimaging can enhance collaboration, accessibility, and replicability (Kiar et al., 2023).

### 4.2 Multi-modal integration and artificial intelligence/machine learning tools

Collecting and integrating data using multimodal methodologies is vital for gaining comprehensive insight into brain development and a deeper understanding of the links between neurobiology, structure, and function (Gabard-Durnam et al., 2018, Bigdely-Shamlo et al., 2015). Simultaneously acquired data can benefit from the strengths of its given methodology whilst overcoming their inherent limitations, such as when combining EEG and fMRI (Arichi et al., 2017, Laufs, 2012). Combining data in this way could also potentially increase diagnostic sensitivity and prognostic accuracy by identifying modes of shared variability across modalities (e.g., patterns that co-occur across EEG, fMRI, or structural MRI measures).

**Fig. 2:**
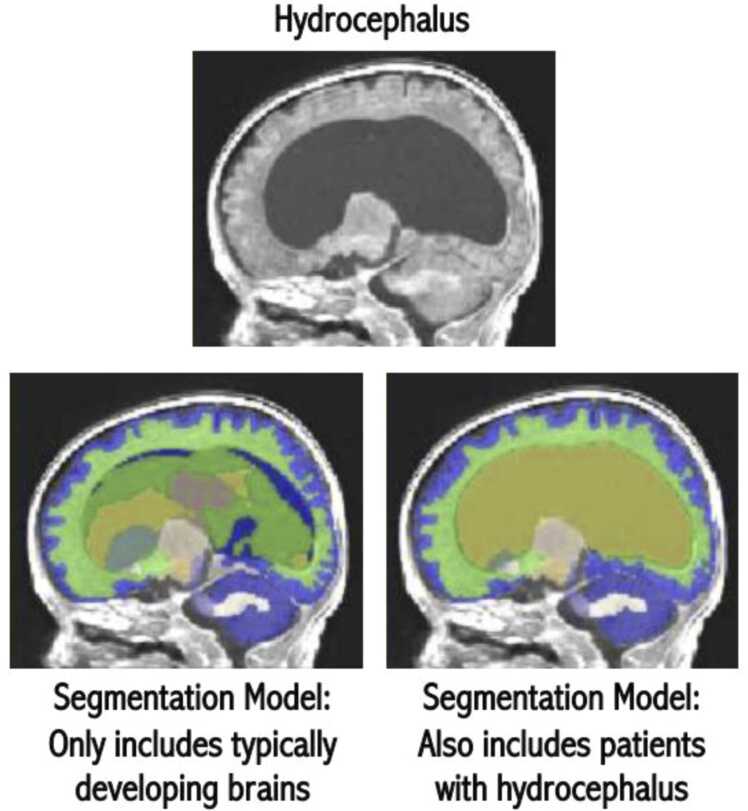
**Automatic segmentation enables accurate volumetric analysis in hydrocephalus.** The images are of a 1-month-old with enlarged ventricles due to hydrocephalus. These differences complicate segmentation pipelines trained exclusively on typically developing brains (Hendrickson et al. 2025). Utilizing the same underlying software while incorporating training images with the brain feature of interest enables automatic segmentations that may require only minimal edits. These segmentations can then provide quantitative measures of brain region volumes for use in clinical practice or research.

The quantitative nature of medical imaging data makes these methods particularly suitable for the application of artificial intelligence (AI) tools, particularly machine learning (ML) methods (Varoquaux and Cheplygina, 2022). The advance of this field has been so rapid and significant that many AI tools are increasingly being certified for clinical use and have the potential to dramatically improve efficiency (Mitsuyama et al., 2025, Pinto-Coelho, 2023). Importantly, such tools can be used to detect subtle differences and deviations that may not be visible through conventional analytical methods, boost image quality (resolution and signal-to-noise), or even generate new data based on existing training data sets (generative AI; Koohi-Moghadam and Bae, 2023). Models can also be retrained for application to vulnerable clinical populations where acquiring large amounts of data is challenging. For example, the deep neural network BIBSNet (Baby and Infant Brain Segmentation Neural Network), an open-source, community-driven model for robust and generalizable brain segmentation leveraging data augmentation with a large sample size of manually annotated images (Hendrickson et al., 2025), can be easily retrained for FIT clinical populations, such as those with hydrocephalus (Fig. 2). However, important considerations about the wider application of AI-based methods remain, particularly with respect to issues including data privacy, applicability to under-represented populations, regulation, and transparency of performance (Herington et al., 2023).

### 4.3 Emerging technologies and industry collaboration

Leveraging cutting-edge technologies, such as cost-effective and bedside neuroimaging solutions, can significantly advance translational FIT neuroimaging research. Portable neuroimaging technologies, for example, have the potential to revolutionize the field by making bedside neuroimaging feasible (e.g., in NICUs and PICUs) and by reaching populations previously excluded from FIT neuroimaging research due to accessibility challenges in under-resourced global settings (Margolis et al., 2025).

Ultrasound stands out as a promising modality for more scalable research applications (Nelson and Narula, 2013). For instance, most fetal and postnatal ultrasounds used in hospital settings cost approximately $50,000 USD, whereas portable ultrasound systems have become available for as low as $2000 USD (Tang et al., 2022). The latter systems, however, currently have several limitations, including shorter battery life and lower image quality compared to hospital-grade systems. Enhancing this technology and making it widely available could impact the scalability of global FIT neuroimaging research, enabling clinicians to assess the presence and extent of the range of mild to severe brain insults in time-sensitive instances. For example, portable ultrasound machines could be used promptly before and immediately after transporting fragile, extremely low birth weight neonates to assess the incidence of germinal matrix-intraventricular hemorrhage, either between hospitals or from a rural hospital to a facility with a higher-level NICU (Browning Carmo et al., 2017). Additionally, they can be utilized within a hospital when extremely low birth weight neonates on ventilators cannot be safely moved for MRI or computed tomography scanning elsewhere, necessitating bedside neuroimaging (Burdjalov et al., 2002).

MEG technology has also seen recent significant advancements, primarily driven by the transition from traditional cryogenic MEG systems, which require cooling to approximately 4 Kelvin (-269°C), to Optically Pumped Magnetometers MEG (OPM-MEG) systems that operate at room temperature (Brookes et al., 2022, Corvilain et al., 2025b, Feys et al., 2022). The main advantages of OPM-MEG include higher experimental flexibility (i.e., wearable OPM sensors can be adapted to various head sizes and have a good head movement tolerance) and comparatively lower upfront and operating costs, with moderate infrastructure requirements compared to cryogenic MEG (e.g., Brookes et al., 2022; Pedersen et al., 2022). Owing to its greater flexibility and on-scalp sensor placement compared to conventional Superconducting Quantum Interference Device MEG (SQUID-MEG), OPM-MEG is becoming increasingly comparable to EEG in developmental research. While both MEG and EEG capture electrophysiological activity with millisecond temporal resolution, their sensitivity differs with cortical maturation. Unlike EEG, OPM-MEG measures magnetic rather than electric fields and is less sensitive to variations in skull conductivity, enhancing spatial precision and source localization accuracy across developmental stages (Boto et al., 2018, Brookes et al., 2022). The main limitations of OPM-MEG remain the need for magnetic shielding, which currently constrains the naturalistic and ecologically flexible developmental paradigms achievable with EEG. Overall, the combination of high spatial accuracy and increased developmental suitability – potentially extending to fetal stages (Corvilain et al., 2025a) not accessible with EEG – makes OPM-MEG a promising tool for developmental research. The clinical utility of OPM-MEG systems is being further investigated, with some adult research suggesting that it is a promising tool that surgeons can rely on for precise localization of tumors (Sun et al., 2024). Low-density OPM-MEG systems (e.g., 32 channels) have also been shown to have comparable accuracy to conventional cryogenic MEG in certain contexts, such as the detection of interictal epileptiform discharges in pediatric populations (Feys et al., 2022).

Substantial progress has also been made in the development of portable, low-resolution MRI machines that are more affordable than conventional static high-field systems. For instance, the ultra-low field Hyperfine Swoop neuroimaging system (64mT) is U.S. Food and Drug Administration approved, does not require a shielded room, and can be operated using a standard electrical outlet. The feasibility of using such technology safely in the NICU has been shown (Sien et al., 2023, Sabir et al., 2023), while providing the highest potential for diagnosing extensive structural alterations in the brain, such as ventricular enlargement (Gruber et al., 2025). While the Hyperfine Swoop system is constrained by reduced SNR, leading to low-resolution images compared to conventional MRI, ongoing research exploring the pairing of Hyperfine and 3 T data acquisition may facilitate enhanced neuroanatomical visualization in low-resolution and low-contrast images (Cawley et al., 2023).

EEG and fNIRS also offer portable and relatively affordable options, aimed at enhancing scalability. Lower-density EEG systems with up to 32 electrodes are available for several hundred to a few thousand US dollars, making them potentially better suited for low- and middle-resource settings (Duncan and Rae, 2024), while high-quality EEG labs or hospital systems can cost $100,000 USD or greater. More research and development are required to continue to improve portable and affordable EEG systems and make higher-density EEG systems more accessible globally (Margolis and Nelson et al., 2025). Lower-cost fNIRS systems, including prototype cost-effective NIRS systems as low as $50 USD (Wu et al., 2022), are starting to emerge and warrent further investigation, as they are increasingly important for studying early cortical development in low- and middle-resource settings.

Lastly, it is equally important to acknowledge that significant technological advancements are being achieved in cutting-edge FIT neuroimaging modalities where availability, affordability, and accessibility remain challenging. Combining state-of-the-art neuroimaging techniques with more cost-effective, portable, and wearable neuroimaging solutions offers a realistic and promising pathway for expanding FIT neuroimaging methods into regular clinical practice. Industry partnerships are also essential for improving scalability, gaining regulatory approval, and encouraging broader dissemination, especially with hardware developers, even though the initial implementation of these innovations may paradoxically raise costs. When combined, these strategies present a forward-looking approach to bridging the gap between cutting-edge FIT neuroimaging innovation and practical clinical application.

### 4.4 Integrating FIT neuroimaging research in clinical care through cross-disciplinary collaborations

**Fig. 3:**
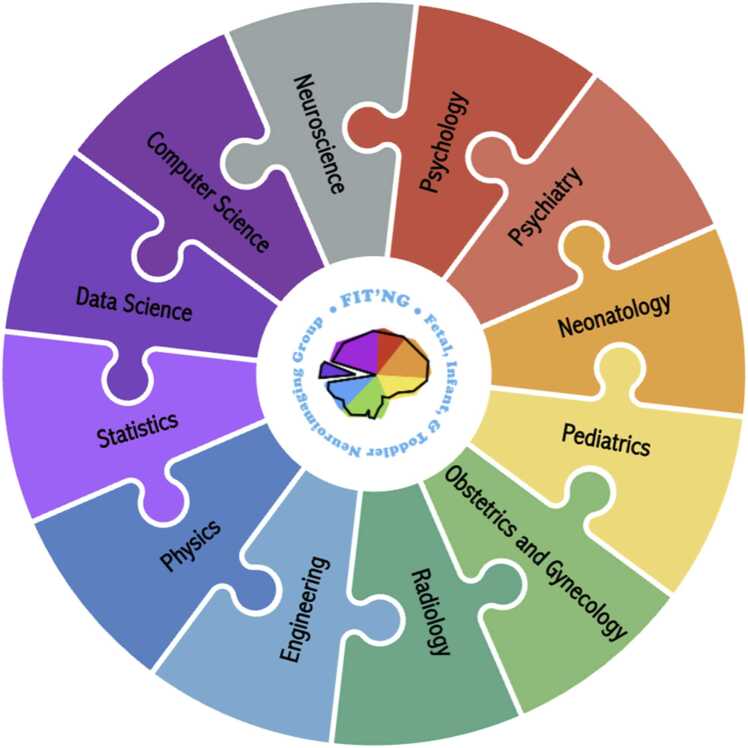
Promoting cross-disciplinary collaboration in fetal, infant, and toddler neuroimaging. FIT’NG aims to provide a forum for early childhood neuroimaging researchers, including those who have technical expertise (e.g., engineers, physicists) and applied researchers (e.g., psychologists, psychiatrists, neonatologists). Reproduced and adapted with permission from Pollatou, A., Filippi, C. A., Aydin, E., Vaughn, K., Thompson, D., Korom, M., Dufford, A. J., Howell, B., Zöllei, L., Di Martino, A., Graham, A., FIT’NG Group, Scheinost, D., & Spann, M. N. (2022). An ode to fetal, infant, and toddler neuroimaging: Chronicling early clinical to research applications with MRI, and an introduction to an academic society connecting the field. *Developmental Cognitive Neuroscience, 54*, 101083. https://doi.org/10.1016/j.dcn.2022.101083.

For FIT neuroimaging studies to be effectively translated into clinical care, the continuously evolving field needs to prioritize establishing shared goals. For instance, these may include neurological biomarkers, standardized data sharing, validated predictive models, patient-centered outcome measures, clear terminology, and harmonized protocols across disciplines. Cross-disciplinary collaboration as further illustrated in Fig. 3 should be prioritized and implemented early in the study design phase through co-design initiatives aimed at crafting rigorous and ethically sound research questions that can enable early and accurate diagnoses and prognoses, eventually helping clinicians to develop individualized treatment plans. Furthermore, to bridge knowledge gaps, efforts such as joint research planning frameworks (e.g., co-designed study protocols and variable definitions), integrated data pipelines (e.g., shared data standards and interoperable analytic tools), and continuous feedback loops (e.g., researcher-clinician liaisons) need to be established.

One of many ways to establish an even stronger bidirectional relationship between researchers and clinicians while simultaneously enhancing the relevance and breadth of translational FIT neuroimaging research alongside its clinical utility, is by embedding FIT neuroimaging research within existing clinical infrastructures. This approach enables the field to move beyond cross-sectional examination of underlying mechanisms and towards causal, more developmentally and clinically informed, individual-level predictions, while better representing the natural heterogeneity of patient populations. To illustrate, the Eunice Kennedy Shriver National Institute of Child Health and Human Development Neonatal Research Network is a federally funded collaborative network of NICUs across the United States comprised of around 15–18 academic institutions, all engaged in multi-center clinical trials and observational studies to assess morbidity and mortality trends over decades. The Neonatal Research Network has validated multiple therapies through multicenter randomized controlled trials, including whole-body hypothermia for HIE and inhaled nitric oxide for hypoxic respiratory failure (Watterberg et al., 2022). In addition, the Neuroimaging and Neurodevelopmental Outcomes (NEURO) study is the most extensive prospective study linking serial postnatal ultrasounds and near-term equivalent MRIs to neurodevelopmental outcomes at 18–22 months corrected age among a cohort of extremely preterm infants participating in a Neonatal Research Network clinical trial (Hintz et al., 2015). This study is one of many that demonstrates the successful integration of FIT neuroimaging research in a clinical setting and was so successful that the same cohort was followed into early school age to assess long-term cognitive outcomes (Hintz et al., 2018). Overall, these clinically integrated studies highlight how FIT neuroimaging-derived insights can be more readily integrated into clinical decision-making, pending appropriate validation, to support a more rapid and scalable path from research to clinical impact.

### 4.5 Developing normative brain development baselines

Enhancing the prioritization of FIT neuroimaging research on age-specific normative brain development is another essential strategy to establish clinical baselines that can help identify atypical growth patterns, particularly in high-risk individuals or cohorts at risk of delays or altered neurodevelopmental trajectories. To illustrate, our understanding of normative brain development in the last trimester is considerably limited, prompting the field to rely on studying preterm cohorts as proxies or indirect measures of fetal brain development. However, the environment of a fetus in late gestation and a preterm infant is drastically different (DeMaster et al., 2019, Flake, 2022, Matthews et al., 2018, McGowan and Vohr, 2019). While a fetus is exposed to a rhythmic auditory environment produced by maternal organs, heart, and voice, a preterm infant’s sensory environment is usually irregular and noisy (Lahav, 2015). Preterm infants also often exhibit additional early medical risks, including bronchopulmonary dysplasia and retinopathy of prematurity, and may require early interventions, such as postnatal corticosteroids that may significantly impact neurodevelopment -- risks not yet present in age-equivalent fetuses (Cuzzilla et al., 2018, Thompson et al., 2012). Technologies that enable the direct study of brain function in fetuses, such as fetal fMRI (e.g., van den Heuvel and Thomason, 2016) or fetal MEG (e.g., Eswaran et al., 2007), are continually evolving, providing better insights into normative fetal development. The future development of these technologies (e.g., OPM-MEG) or improvements in motion correction for fetal MRI will help facilitate direct studies of normative brain development in the last trimester of gestation.

Recent work is beginning to fill these normative gaps. Scheinost et al., (2022) provided one of the first mappings of functional network maturation from the third trimester to the early newborn period (up to ∼44 weeks post menstrual age), focusing on the default mode, frontoparietal, and salience networks. They demonstrated distinct network trajectories and showed that maternal stress can modulate these trajectories. Building on this, Ji et al. (2024) documented maturational changes across the fetal-to-newborn transition using a broader, whole-brain network approach. By examining shifts in whole-connectome properties over a wider age range (∼ 25–55 weeks post menstrual age), they identified dramatic reorganization at birth in select networks (e.g., occipital-cerebellar, subcortical, and superior frontal), alongside more gradual increases in other networks (e.g., sensorimotor). Collectively, these studies help define developmental trajectories of functional connectivity across the perinatal period, providing baselines for typical development and improving understanding of early brain development. If the field shifts toward using more robust and flexible frameworks (e.g., GAMLSS; Bethlehem et al., 2022; Rigby and Stasinopoulos, 2005) to model typical and atypical neurodevelopmental trajectories in large samples across the FIT continuum, insights may become more effectively translatable into clinical practice while still taking into account individual variability (Olson et al., 2025).

## 5 The path forward: framework for translation and future directions

Building on the FIT neuroimaging modalities discussed throughout this review, we provide a concise summary table outlining the current clinical utility of various neuroimaging techniques used in FIT populations. This non-exhaustive list includes only those modalities covered in the main text and highlights examples where clinical applications have already been demonstrated. The information in Table 2 represents a current snapshot of each modality’s translational status, describing their general characteristics, benefits, limitations, and validated clinical uses. To enable comparison across modalities, a *Clinical Readiness Range* is included, scored from 1 to 4. This scale reflects the extent to which each modality has been validated for clinical use: (1) technologies primarily used in basic science or research settings with minimal clinical integration; (2) emerging tools with some evidence of clinical relevance but lacking sufficient validation; (3) modalities with demonstrated clinical utility in specific contexts but requiring broader evidence for widespread adoption; and (4) techniques that are established and suitable for routine clinical application. This framework is intended to provide clinicians and researchers with a high-level view of where each modality currently stands in the translational pipeline. Lastly, this review does not prioritize specific strategies; it is intended as a self-reflective discussion to generate conversations about how FIT neuroimaging research and insights can be applied beyond academic settings to enhance clinical outcomes. We anticipate that the field will come together to identify priorities and refine approaches for implementing these strategies to improve pediatric healthcare and developmental outcomes.

**Table 2:** Examples of clinical relevance and implementation readiness of key neuroimaging modalities in fetal, infant, and toddler research.

**Modality**	**Information Provided**	**Benefits**	**Limitations**	**Examples of Validated Clinical Applications**	**Examples of Emerging Clinical Potential**	**Clinical Readiness Range**
**Magnetic Resonance Imaging (MRI)**						
Diffusion MRI	Delineation of white matter pathways Tissue microstructure	No ionizing radiation Higher sensitivity to acute phases of tissue damage Detailed maps of whole brain structural connectivity	Cost-inefficient Low global density and availability of technology necessary for data collection Contraindications (e.g., metal in body) Susceptible to artifacts (e.g., echo-planar imaging [EPI] artifacts, motion, metal) Noise reduction necessary during data collection Restricted sequence parameters due to potential heating from high specific absorption rate (SAR)	Identification of tissue injury due to ischemia (e.g., decreased apparent diffusion coefficient [ADC] values) Neurosurgical pre-operative planning (e.g., mapping of neural pathways to avoid functional impairment arising from surgical resections)	Assessing tissue damage in HIE (Onda et al., 2022) Predicting Autism Spectrum Disorder severity (Kilroy et al., 2022) Detecting divergent developmental trajectories (DiPiero et al., 2023) Improving non-invasive diagnostic certainty in neurooncology (Grist et al., 2020)	3
Functional MRI	Indirect measure of neural activity (Blood Oxygen Level Dependent [BOLD] changes) and connectivity during task and rest	No ionizing radiation Enables whole brain mapping of functional connectivity	Cost-inefficient Low global density and availability of technology necessary for data collection Contraindications (e.g., metal in body) Sensitivity to artifacts (e.g., echo-planar imaging [EPI] artifacts, motion, metal) Noise reduction necessary during data collection Sleep state-related variability in brain activity	Altered functional connectivity demonstrated in infants at risk of neurodevelopmental conditions (e.g., cerebral palsy)	Surgical planning (Charbonnier et al., 2020, Jones et al., 2020) Predicting autism spectrum disorder diagnosis with familial predisposition (Emerson et al., 2017) Predicting cognitive impairments in cerebral palsy (Moll et al., 2021) Amygdala functional connectivity and negative reactive temperament associations, as a marker of anxiety risk (Filippi et al., 2021)	2
Structural MRI	Anatomical delineation of cortical and subcortical structures Measurement of tissue volumes and regional morphology, such as cortical thickness, surface area, and gyrification	No ionizing radiation Excellent spatial resolution	Cost-inefficient Low global density and availability of technology necessary for data collection Contraindications (e.g., metal in body) Sensitivity to artifacts (e.g., echo-planar imaging [EPI] artifacts, motion, metal) Noise reduction necessary during data collection Restricted sequence parameters due to potential heating from high SAR	Identification of acute tissue injury Characterization of altered brain growth and development due to congenital or acquired pathology	Neuromotor outcomes and structural correlates in hemiparesis after neonatal ischemic stroke (Boardman et al., 2005) Fetal exposure to HIV immunosuppressants and neonatal structural brain volume (Wedderburn et al., 2022) Predicting motor development outcomes at 9 months of age using structural MRI in neonates with critical congenital heart disease (Stegeman et al., 2022) Early-stage ASD status prediction (Gao et al., 2021)	4
Magnetic Resonance Spectroscopy (MRS)	Measurement of brain metabolite levels and the presence of abnormal/altered neurochemicals Identification of raised lactate indicating ischemic tissue injury	No ionizing radiation	Poor spatial resolution Sensitivity to artifacts (e.g., motion, tissue partial voluming) Noise reduction necessary during data collection	Increased lactate:NAA ratio predicts injury severity and neurodevelopmental outcome in HIE	Early prediction of neurodevelopmental outcomes (Mitra et al., 2019) Prognostic value of lactate/N-acetylaspartate ratio in neonatal encephalopathy (Barta et al., 2018) Association of thalamic metabolite ratios with white matter injury (Montaldo et al., 2020)	4
Portable Ultra-Low Field MRI	Anatomical delineation of cortical and subcortical structures Measurement of tissue volumes and regional morphology	Cost-efficient No ionizing radiation Point of care imaging in clinical practice (e.g., NICU) Potential for use in low-income settings	Low spatial resolution compared to high-field MRI Weak magnetic field hinders reliable fMRI and MRS detection	Identification of acute tissue injury Characterization of altered brain growth and development due to congenital or acquired pathology	Assess ventricular size and morphology in cases of hydrocephalus (Gruber et al., 2025)	2
Magnetic Resonance Angiography (MRA)	Visualize blood vessels; some sequences provide information about blood flow	No ionizing radiation No contrast agent needed	Visualizes flow as opposed to true vessel anatomy Low spatial resolution Long scan time May require sedation in infants	Assess congenital vascular malformations (e.g., vein of Galen malformation) Detects cerebral venous sinus thrombosis	Identification of infants at risk for childhood arterial ischemic stroke (Fullerton et al., 2007)	4
**Magnetoencephalography (MEG)**						
MEG	Measurement of the magnetic fields generated by electrical currents flowing through neurons Direct measure of neural activation	No ionizing radiation Millisecond temporal resolution Silent data collection Short setup time Can be used for fetal imaging Optically Pumped Magnetometers MEG (OPM-MEG) is wearable and is more tolerant of motion than Cryogenic-MEG	Cost-inefficient Limited spatial resolution Expensive technology (Cryogenic-MEG requires helium, OPM-MEG does not), including magnetically shielded room and maintenance Cryogenic-MEG: One-size-fits-all helmet that limits the use in infancy OPM-MEG: Still emerging; validation and standardization are currently limited	Functional mapping for tumor resection planning Detection of interictal epileptiform discharges and pre-surgical mapping in epilepsy	Prediction of ASD severity, and identification of auditory and language impairments (Green et al., 2020, Roberts et al., 2008) Fetal autonomic regulation via magnetocardiography (Escalona-Vargas et al., 2024, Strand et al., 2019) Assessment of fetal brain function: recording of visual and auditory evoked responses from 27 weeks of gestation (Corvilain et al., 2025a, Draganova et al., 2007, Eswaran et al., 2021, McCubbin et al., 2007)	3
**Near-Infrared Spectroscopy (NIRS)**						
Functional NIRS (fNIRS)	Measure of changes in oxygenated and deoxygenated hemoglobin concentrations in channel space	Cost-efficient No ionizing radiation Portable Tolerant to motion Subjects can be awake or asleep Time domain systems can measure absolute tissue concentration	Temporal constraints (hemodynamic response on order of seconds) Limited depth penetration (i.e., can only measure surface cortical activity) Sensitivity to external light, but can be effectively mitigated Hair interference can reduce SNR	Is not currently considered a clinical tool	Bedside monitoring of brain injuries in preterm neonates (e.g., germinal matrix-intraventricular hemorrhage) (Kebaya et al., 2023) Tracking neurodevelopmental trajectories (e.g., language and communication disorders) (Gallagher et al., 2016) Monitoring cerebral oxygenation in preterm infants with hypoglycemia in NICU using diffusion optical tomography (DOT) (Perkins et al., 2025)	2
NIRS	Measure of tissue oxygenation			Monitoring cerebral and/or somatic oxygenation	Time-domain diffuse correlation spectroscopy for relative blood flow index sensitive to cortical tissue (Parfentyeva et al., 2023) Resting-state optical and hemodynamic reference values (Calcaterra et al., 2025)	3
**Electroencephalography (EEG)**						
EEG	Measures electrical potentials and neural oscillations on the scalp, which are generated by the firing of millions of neurons Broadband neural activity that may reflect excitatory/inhibitory balance	Cost-efficient No ionizing radiation High temporal resolution Can be portable Mobile (wireless) systems can facilitate use in naturalistic paradigms	Limited spatial resolution Susceptible to artifacts; motion artifact can be particularly challenging in FIT populations Cannot measure deep brain activity (limited to cortical post-synaptic potentials)	Epilepsy and sleep disorders diagnosis and management Bedside monitoring and treatment management of neonatal encephalopathy Identifying sleep stages Assessing seizures in preterm infants Visual evoked potential used in ophthalmology to diagnosis and monitor disorders associated with the visual pathway Auditory brainstem response is a simplified form of EEG used to test hearing in infants	Clinical trial stratification and biomarker (Goodspeed et al., 2023) Monitoring during anesthesia (Sun et al., 2020) Screening for neurodevelopmental disorders (An et al., 2025, Bauer and Bosl, 2025) Auditory brainstem response: detection of neurobiological abnormalities in the 8th cranial nerve and its related auditory pathways (Young et al., 2025)	4
**Ultrasonography**						
Fetal Ultrasound	Fetal head biometry (e.g., biparietal diameter) Anatomical integrity of brain regions (e.g., ventricular spaces)	Cost-efficient No ionizing radiation Portable, enables bedside imaging	Limited resolution and depth penetration Sonographer-dependent quality	Detecting anatomical abnormalities (e.g., enlarged ventricular spaces) and central nervous system (CNS) anomalies(e.g., ventriculomegaly and agenesis of corpus callosum)	Improved identification of subtle/mild CNS anomalies with high-resolution 3D/4D (Namburete et al., 2023) Using uterine artery (UtA) pulsatility index (PI) values via uterine doppler ultrasound to indirectly measure midbrain growth and cortical development (Mappa et al., 2024)	4
Neonatal Ultrasound	Structural brain imaging Germinal-matrix, intraventricular, and intraparenchymal hemorrhages Ventricular size Echogenicity	Cost-efficient No ionizing radiation Portable, enables bedside imaging Early/serial imaging (e.g., immediately after birth)	Limited resolution and depth penetration Sonographer-dependent quality Closing of acoustic windows and fontanelles Difficult detecting mild atrophy and insults (e.g., punctate white matter lesions)	Screening for preterm neonates Brain growth and maturation Identifying/ruling out moderate-to-severe structural brain abnormalities/injuries Assess timing and progression of injuries Estimate neurological prognosis	Point-of-care 3D head ultrasonography for objective volumetric brain assessments (Benavente-Fernández et al., 2021) Integration of deep learning-based tools to identify structural abnormalities indicative of brain injury (Ahmad et al. 2025)	4
Transcranial Doppler Ultrasound	Measurement of cerebrovascular anatomy (e.g., cerebral arteries, veins, sinuses)	Cost-efficient No ionizing radiation Portable, enables bedside imaging Excellent sensitivity for detecting vasospasm, stroke risk, and cerebral emboli	Limited to basal arteries Closing of acoustic windows and fontanelles Operator dependency	Assessment of cerebral blood flow velocities over time (e.g., abnormal flow patterns) in various clinical contexts (e.g., severe respiratory disease, patent ductus arteriosus) Assessment of cerebral vasculature Information regarding risk and severity of brain injuries	Integration with NIRS for multimodal cerebral monitoring (Mitra et al., 2011) Serial neuromonitoring to track cerebral hemodynamics in neonatal ischemic encephalopathy (Natique et al., 2021)	4

[Table 2]

## 6 Conclusion

Taken together, FIT neuroimaging research shows clear promise for advancing pediatric medicine by supporting earlier and more accurate diagnoses, improving prognosis, and enabling more personalized interventions. However, to fully realize this potential, important translational challenges must be addressed, including predictive limitations, individual differences, methodological and technological constraints, as well as accessibility and global equity issues. Strategies such as creating standardized neuroimaging protocols, utilizing AI and ML, leveraging emerging technologies, and fostering cross-disciplinary partnerships may help overcome these barriers. With continued efforts to bridge the gap between FIT neuroimaging research and clinical practice, the field can enhance outcomes for fetuses, infants, and toddlers affected by neurological injuries and disorders, particularly in early developmental windows where intervention is most impactful.

## Funding

• P.M.N is supported by the 10.13039/100000025National Institute of Mental Health, Iowa Neuroscience Specialty Program in Research Education (UI Institutional Postdoctoral Research Training Program; T32MH019113).
• M.K is supported by the National Institute of Mental Health Intramural Research Program (ZIA-MH002782).
• G.A.P is supported by a European Research Council grant (ERC-2021-STG).
• S.M.S is supported by the National Science Foundation Graduate Research Fellowship Program (NSF-GRFP; #2237827).
• J.M. is supported by the 10.13039/100000025National Institute of Mental Health (R01MH134966).
• C. C. is supported by the Fonds de la Recherche Scientifique (FRS-FNRS, Brussels, Belgium; T.0026.24).
• I.N.H is funded by the 10.13039/100000071National Institute of Child Health and Human Development (NICHD; 5R01HD109436–03).
• T.A. is supported by an MRC UK Senior Clinical Fellowship [MR/Y009665/1] and the MRC Centre for Neurodevelopmental Disorders, King’s College London [MR/N026063/1].
• Angelina Vernetti is supported by the Associates of the Child Study Center (NIH P50 MH115716; NIH R01 MH137609).

## CRediT authorship contribution statement

**Sanjana Inala:** Writing – review & editing, Writing – original draft, Visualization, Conceptualization. **Istvan N. Huszar:** Writing – review & editing, Writing – original draft, Conceptualization. **Paige M. Nelson:** Writing – review & editing, Writing – original draft, Visualization, Supervision, Conceptualization. **Michal R. Zieff:** Writing – review & editing, Writing – original draft, Conceptualization. **Dashiell D. Sacks:** Writing – review & editing, Writing – original draft, Supervision, Conceptualization. **Angelina Vernetti:** Writing – review & editing, Writing – original draft, Conceptualization. **Marta Korom:** Writing – review & editing, Writing – original draft, Visualization, Conceptualization. **Sally M. Stoyell:** Writing – review & editing, Writing – original draft, Visualization, Conceptualization. **Megan E. Evans**​​​​​​​: Writing – review & editing, Writing – original draft, Conceptualization. **Julia Moser:** Writing – review & editing, Writing – original draft, Visualization, Conceptualization. **Chiara Capparini:** Writing – review & editing, Writing – original draft, Conceptualization. **Elizabeth S. Norton:** Writing – review & editing, Writing – original draft, Conceptualization. **Nushka Remec:** Writing – review & editing, Writing – original draft, Conceptualization. **Guy A. Perkins:** Writing – review & editing, Writing – original draft, Conceptualization. **Carol L. Wilkinson:** Writing – review & editing, Conceptualization. **Tomoki Arichi:** Writing – review & editing, Writing – original draft, Conceptualization, Supervision. **Kevin M. Cook:** Writing – review & editing, Writing – original draft, Conceptualization.

## Declaration of Competing Interest

The authors declare the following financial interests/personal relationships which may be considered as potential competing interests: Dr. Carol L. Wilkinson is a consultant for BioMarin.

## Data Availability

This is a review article from the FIT'NG 2024 Special Issue.
